# The Perils of Riding Motocross: A Summary of this Extensive, Prospective Study

**DOI:** 10.1007/s43465-022-00815-0

**Published:** 2023-02-07

**Authors:** Bruce Hay, Rohit Singh, Stuart Hay

**Affiliations:** 1 University Hospital Wishaw, 50 Netherton St, Wishaw, ML2 0DP UK; 2 Royal Shrewsbury Hospital, The Shrewsbury and Telford Hospital NHS Trust, Mytton Oak Road, Shrewsbury, SY3 8XQ UK; 3 The Robert Jones and Agnes Hunt Orthopaedic Hospital NHS Foundation Trust, Oswestry, SY10 7AG UK; 4 Keele University Medical School, Newcastle-under-Lyme, UK

**Keywords:** Motocross, Orthopaedics, Trauma, Hand, Wrist, Paediatric, Spine, Shoulder, Elbow, Foot, Ankle

## Abstract

**Background:**

Motocross is a high-risk form of motorbiking where serious injuries occur regularly, although little data have been collected to illustrate this relationship. Over 5 years, teams from RJAH Oswestry and RSH sought to demonstrate the impact of Motocross on orthopaedic presentation and workload.

**Method:**

Data were collected prospectively over 5 years including 615 orthopaedic injuries associated with both recreational and competitive motocross.

**Results:**

An increase in injury and operation frequency was observed, young males were identified as the highest risk participant. This was evident over winter and weekends, during the competitive racing season. A variety of injuries have been implicated, some with life threatening or disabling consequences.

**Conclusion:**

Motocross has seen exponential growth in popularity with increases in injuries and operations. This implicates major impacts on finances and healthcare, especially at times of seasonal vulnerability. The authors encourage event organisers to explore the avenues of rider safety in this increasingly popular sport.

## Introduction

Motocross is a sport of increasing global popularity. With its origin in UK motorbike racing during the early 1900s, the discipline involves the negotiation of challenging off road terrain while travelling at speeds of up to 90 miles per hour. In the UK alone, some 200 motocross clubs exist giving rise to over 900 annual racing events [1] attracting participants across the age-spectrum.

As riders push themselves and their vehicles to the limit, there is considerable risk of injury, varying from mild musculoskeletal damage to life-threatening polytrauma. Despite its prominence as an adrenaline-sport, little data exists to explore the epidemiology of associated orthopaedic injuries. Having identified an absence of representative data, Orthopaedic teams from the Robert Jones and Agnes Hunt Orthopaedic Hospital, Oswestry, and the Royal Shrewsbury Hospital conducted a prospective study of the Orthopaedic injury patterns observed in Motocross. A number of epidemiological findings were made and these have been presented across several subspecialties. This aimed to provide data to authorities responsible for planning trauma resources at motocross events, and assist the sport’s governing body ACUBM in their approach to safety through the development of protective equipment, improvement in licensing laws and coaching to develop rider technique.

This article presents a summarised overview of this data, providing perspective and context to the patterns of injury recorded.

## Method

Each patient who attended fracture clinic was analysed including their individual “mechanism of injury”. The details on any injuries associated with off road motorcycling, both recreational and competitive, were sent to the senior author at initial presentation and throughout their management. Data was collected prospectively and added to a database. Initially conclusions were drawn from a 4 year cohort comprising 423 motocross riders, but the study was subsequently extended for a further year and the other articles therefore refer to a total of 615 injuries. Articles based on this series of patients have addressed specific orthopaedic subspecialties. For clarity, the important findings of these articles has been summarised.

## A Prospective Analysis of 423 Injured Riders

An earlier analysis of 423 motocross riders was based on data collected prospectively over a 4 year period [2]. All injuries caused by recreational and competitive riding which were referred to the Trauma Orthopaedic Department were included.

Overall, injuries were more commonplace in competition riders than recreational, with an overwhelming majority of male racers (88%) compared with female (12%). The largest cohort belonged to 12–20-year-olds, although age ranged from 3 to 73.

Interestingly, seasonal differences were also detected—demonstrating periods where riders were more susceptible to injury. Predominantly, the months of March and April were implicated—with this corresponding to the start of the Motocross season. To a lesser extent, a peak was also witnessed between October and November, we believe related to more treacherous conditions of both circuit and weather at this time of year.

A total of 485 injuries were recorded over five years, and varied from isolated injuries to 6 injuries per patient. Eighteen percent of patients suffered at least two injuries, with some including significant polytrauma events or life-threatening injuries to the head and spine. These required transfer to specialist centres for neurosurgical, spinal and cardiothoracic input.

Almost half of the 423 patients required operative intervention on account of their injuries, with 12% requiring multiple procedures and prolonged hospital admissions. These operative figures and injury frequencies increased exponentially over the four years, mimicking global trends [3].

**Fig. 1:**
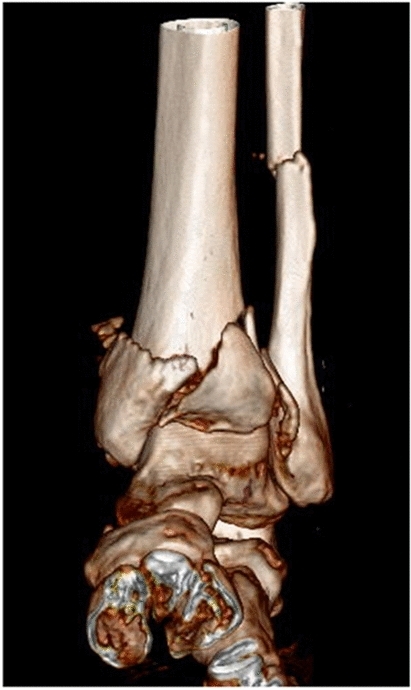
CT three-dimensional reconstructed coronal image of a comminuted distal tibia-fibula fracture of the left lower limb

Various injury patterns were observed, with 15 different categories of injury classified in the paper. The frequencies of these injuries was spread relatively evenly, with the most common of these—clavicular fracture accounting for 14% of the total. The next most common injuries—long bone fractures, forearm/distal radius fracture and shoulder injuries accounted for 11%, 9.7% and 8.9% respectively (Fig. 1).

## An Epidemiological Study of Paediatric Motocross Injuries in the United Kingdom

Using the same data, paediatric motocross injuries and outcomes were categorised and quantified [4]. This analysis concerned those aged 3–17, and comprised 130 patients and a total of 142 injuries occurring in both recreational and competitive motocross. Annually, this was shown to increase, a pattern also identified with the adult data. A total of 18% suffered two injuries or more, with 8 cases requiring emergency High Dependency or Intensive Care admission. Four cases involved serious head injury requiring neurosurgery, all of which survived, but require full time care. Additionally, two patients required transfer for urgent Cervical Spine stabilization for fracture dislocation, while two other patients required Cardiothoracic transfer following haemo/pneumothorax.

Male competitive racers were at highest risk as in the previous cohort and these represented 92%. Interestingly, no significant variation in injury-type amongst different age groups within this cohort was identified. However, seasonal variation was observed once again, with the spring and winter months showing the highest injury frequency. Of the 130 patients included in this cohort, 76 required admission and 60 required surgery. A number of these required multiple procedures and had longer admissions to hospital representing an average of 2 days per patient.

Injury patterns were again varied amongst the paediatric cohort—the three most common patterns being clavicle fractures (14%), forearm/distal radius fractures (13%) and long bone fractures (12%). Note was also made of other significant paediatric presentations. Four physeal injuries of distal femur were observed, and one patient experienced subsequent leg length discrepancy. A further five patients sustained significant soft tissue knee injuries; with cruciate ligament and meniscal injuries implicated in these cases. Additionally, 4 Gartland type III supracondylar humeral fractures were seen, each requiring closed reduction and pinning.

## Shoulder and Elbow Injuries Related to Motocross: A 5-Year Review

This article analysed a series of adult and paediatric patients collected over five years, and concentrated specifically on injury patterns involving the shoulder and elbow [5]. Amongst this cohort, 205 patients were included, sustaining a total of 240 injuries ranging from 1 to 4 per patient. The five-year trends showed that both the number of injuries and the number of operations performed had increased annually. Demographic findings remained identical, with male competition riders from 11 to 20 years of age being at highest risk. Injuries were more common in spring and winter months.

**Fig. 2:**
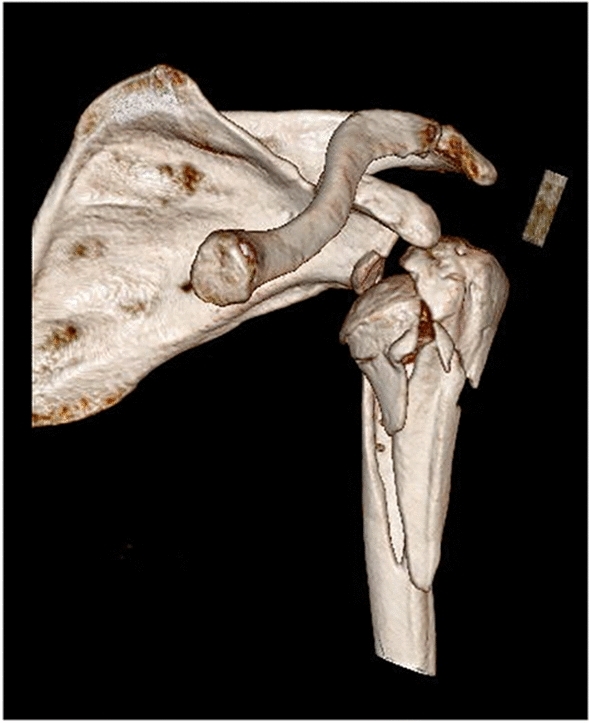
CT three-dimensional reconstructed coronal image of a right sided comminuted proximal humeral and glenoid fracture

**Fig. 3:**
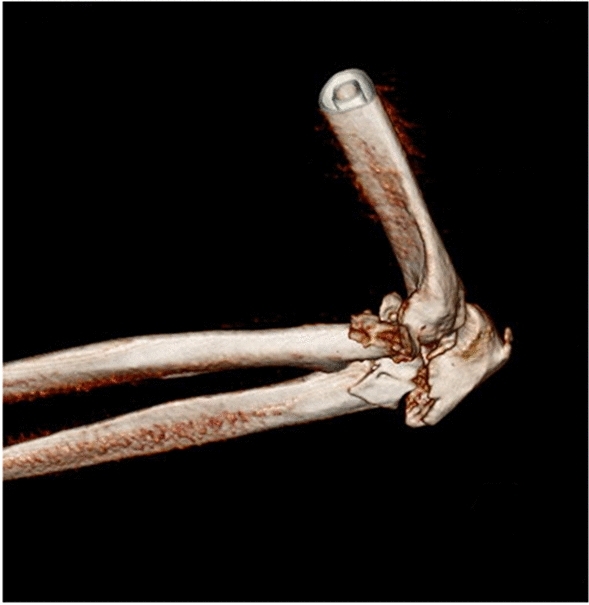
CT three-dimensional reconstructed sagittal image of a right sided comminuted proximal ulna and radial head fracture

Amongst this cohort, the most common injury pattern was Clavicle fracture (19%) followed by Elbow dislocation (10%) and olecranon fracture (9.5%). Fifty per cent of patients required in-patient hospital stay, ranging from 1 to 10 days with a mean stay of 2 days (Figs. 2, 3).

## Spinal Motocross Injuries in the United Kingdom

With respect to spinal injuries in Motocross, the demographic findings were again comparable, with male competitive Motocross riders implicated more than both recreational and female riders. The same seasonal differences were evident in frequency of spinal injuries, with spring and winter months showing a peak incidence of injury [6].

The cohort included 174 patients presenting with 216 injuries, ranging between 1 and 6 per patient. This included several orthopaedic pathologies, although associated rib and visceral injury was found to be the most frequent presentation with 128 injuries in 110 of the patients included. Of the spinal specific injuries, thoracolumbar burst fractures (44%) and chance fractures (12%) were the most common.

A total of 54% of injuries needed operative intervention, requiring a 7-day hospital admission on average. Many of these cases required HDU/ICU admission (25%), and 11% proceeded to emergency laparotomies. The frequency of injury was found to increase annually with an increase of 500% noted during the 5-year period.

The chronic and debilitating nature of these conditions was also highlighted, with emphasis placed on associated complications. Many patients who were referred suffered major life threatening and or permanently disabling complications; requiring continued nursing care and rehabilitation often indefinitely after admission. “Return to work” occurred in just 31% of this group which was noted to be much lower than other studies had previously suggested.

## An Epidemiological Study of Foot and Ankle Motocross Motorcycling Injuries in the United Kingdom

**Fig. 4:**
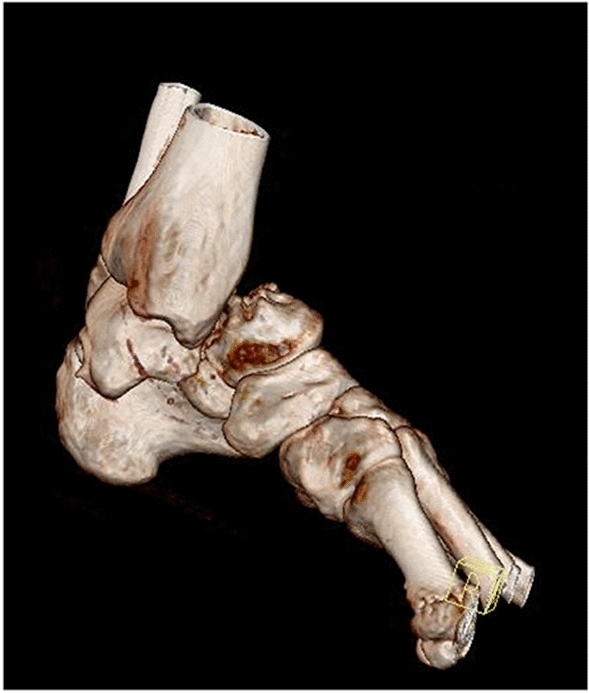
CT three-dimensional reconstructed sagittal image of a left sided displaced navicular and cuboid fracture with talo-calcaneal dislocation

In this study, 235 foot and ankle injuries were collected over the five-year period [7]. The demographic trends remained similar with 21–30-year-old male competitive riders reporting a higher incidence of injury, especially in the spring months (Fig. 4).

**Fig. 5:**
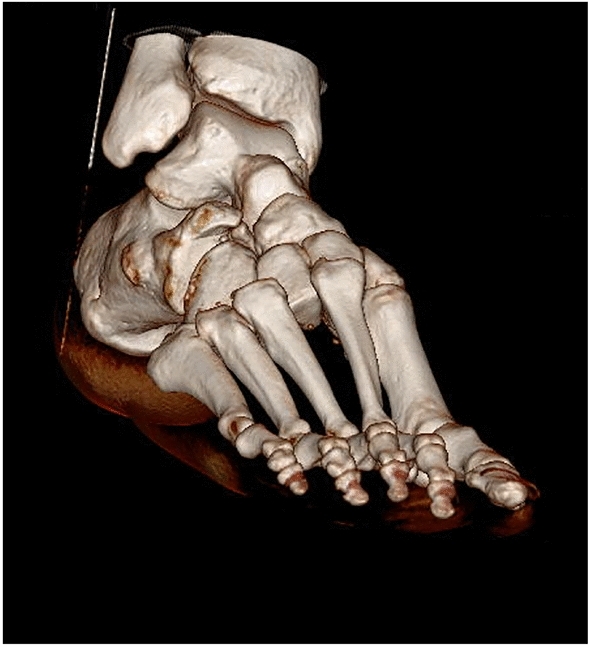
CT three-dimensional reconstructed coronal image demonstrating tarsel-metatarsal dislocation of the second, third, fourth and fifth metatarsals

The most common injury encountered were ankle fractures making up 23% of the total. This was followed by ankle sprain (21%) and metatarsal fractures (17%). Most injuries required only conservative management, but a total of 36% of patients required operative intervention. The injury frequency and number of operations again were shown to increase over the five-year period (Fig. 5).

## Hand and Wrist Injuries Related to Motocross Injuries: 5-Year Series

A total of 265 hand and wrist injuries were observed across the 5-year study period, involving 240 patients [8]. Male competitive riders were predominantly implicated, with most injuries occurring in 11–20-year-old patients. Seasonal peaks in the spring and winter months were again noted. Of the 240 patients included, 40% required operative intervention, some requiring multiple procedures. A wide spectrum of injuries was seen, comprising mainly of distal radius injuries (20%), followed by metacarpal fractures (14%) and phalangeal fractures (13.5%). Again, the trend showed a rise in both injury frequency and procedures performed annually.

## Discussion

Motocross involves off road motorcycling, travelling at high speeds on treacherous terrain, sometimes recreationally, but often with 25–30 riders simultaneously competing during a single race. It is a sport of increasing popularity, both recreationally and competitively. Over the last decade, the number of annual tournaments and racers within the UK has doubled, with similar trends seen worldwide [9, 10]. It attracts a huge age range, with children as young as 3 and adults as old as 73 competing [2]. It is easily accessible for both adults and children. The UK’s Amateur Motor Cycle Association allows competitors as young as 6 to apply, with an online license application and payment of an entrance fee being the only pre-requisites for competitive racing [1].

It is by nature, regarded as a high-risk sport. Vehicles are capable of speeds of 90 mph, and subsequent collisions with terrain or other participants occur with high-energy impact. Indeed, significant and sometimes life-threatening injuries occurring as a result of motocross have previously been documented [11–13]. However, this analysis of a five-year sequential data series is the first of its kind and demonstrates conclusively both the magnitude and the variety of orthopaedic injuries incurred.

Measures to reduce the risks during races include the use of protective equipment, as well as onsite health and safety management, fire safety management, crisis management planning, safe working practice documents and pedestrian management solutions [1].

Despite these measures, all cohorts analysed within this series demonstrate an increasing incidence of injury and subsequent operative procedures annually. These injuries are multi-faceted often with devastating consequence, particularly when considering the age of most participants. Nearly a fifth of the 423 patients included in the first report suffered two or more injuries and several required transfer to tertiary centres for the management of life-threatening neurosurgical, spinal or cardiothoracic complications [2].

The wider economic and seasonal burdens of motocross on hospitals also warrant consideration. This study has shown that in spite of attempts to reduce the injury rate in motocross, a significant rise in both injuries sustained and operations performed is evident. When considering the cost of an in-patient bed, surgery and rehabilitation, delayed return to work, loss of taxes and the funding of patient support networks, the cost burden of injuries resulting from Motocross is considerable.

As demonstrated, injuries tend to occur more often in competitive events, which necessarily take place at weekends when participants are available. Furthermore, there is a clear seasonal pattern of injury, with spring and winter months heavily implicated. Weekends and winter in particular are the times when hospital resources are often stretched and non-more so than in recent Winters, given the considerable demand of the Coronavirus Pandemic. Request has been made to encourage that competitions take place within the vicinity of trauma centres, although as these studies clearly demonstrate, smaller district hospitals are often implicated. These extra numbers will inevitably impact on the efficiency of service offered to other patients and should be given appropriate consideration in healthcare planning.

## Conclusion

Motocross is a high adrenaline motor-sport across unpredictable terrain and inevitably results in high-energy impact injuries. Orthopaedic trauma units receiving casualties from this sport will recognise the impact that this has on resources, and the importance of preparing for this challenge since “fore-warned is fore-armed”.

**Fig. 6:**
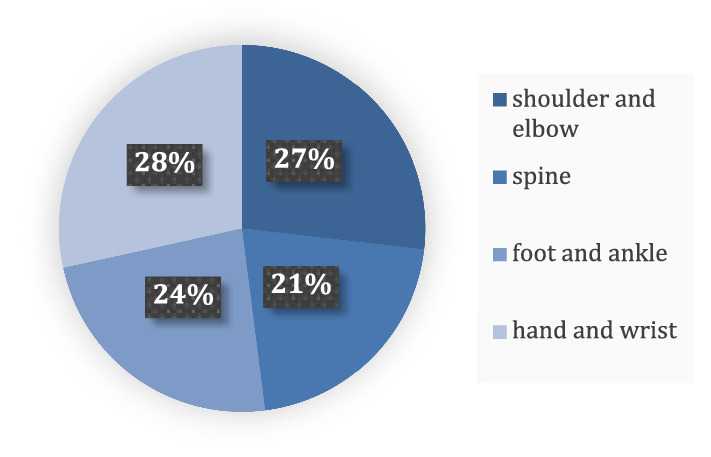
Pie Chart showing percentage distribution of Motocross Injuries between 2010 and 2015

The authors therefore established this analysis of motocross injuries—the first study of its kind. After 5 years the cohort was large enough to meaningfully categorise the associated injury patterns, including various Orthopaedic subspecialties (Fig. 6).

Seasonal and demographic patterns were recognised, with young male competitive racers at higher risk of injury. Injuries tended to take place in either spring months coinciding with the start of the motocross season or in winter months as conditions became more treacherous.

**Fig. 7:**
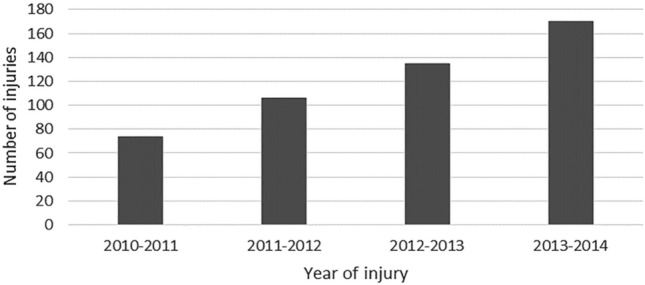
A Bar Chart demonstrating the annual increase in injury frequency of all types caused by Motocross over the study period

**Fig. 8:**
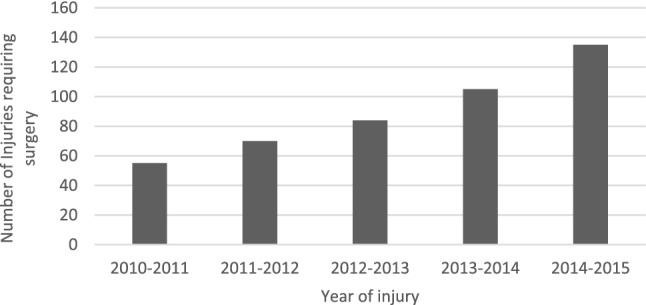
A Bar Chart showing the total number of motocross injuries requiring surgical treatment between 2010 and 2015

Injury frequency and operation numbers increased over the five-year period, creating significant economic burden for hospitals and health implications for participants. A spectrum of injury severity ranged from minor to life threatening (see Figs. 7, 8).

Given the growing popularity of motocross across the World, this study strongly suggests an emphasis on safety measures. Some initiatives might include safer individual course design, restrictions on competitor age, restriction on the number of race participants, immediate availability of appropriate healthcare professionals and an emphasis on protective clothing. Such initiatives might be facilitated through a collaboration between the local event organisers and ACUBM, potentially improving safety/outcome for both participants and spectators.

It is also clear that each healthcare provider must allocate sufficient resources including appropriate manpower and equipment, to accommodate the surges in activity associated with these events and their related injuries.

## Data Availability

The authors confirm that all data referred to in this summary is available within the article, its supplementary materials and its references.

## Declarations

### Data sharing

NA.
